# Effects of an intervention targeting social media app use on well‐being outcomes: A randomized controlled trial

**DOI:** 10.1111/aphw.12646

**Published:** 2025-01-24

**Authors:** Lina Christin Brockmeier, Lea Mertens, Christina Roitzheim, Theda Radtke, Tilman Dingler, Jan Keller

**Affiliations:** ^1^ Department of Education and Psychology, Division of Health Psychology Freie Universität Berlin Berlin Germany; ^2^ Faculty of Psychology and Neuroscience Maastricht University Maastricht Netherlands; ^3^ Wellspent GmbH Berlin Germany; ^4^ Department of Health Psychology and Applied Diagnostics University of Wuppertal Wuppertal Germany; ^5^ Department of Sustainable Design Engineering Delft University of Technology Delft Netherlands

**Keywords:** affect, digital disconnection, digital nudge, intervention app, problematic smartphone use, stress

## Abstract

**Background:**

Interventions targeting social media use show mixed results in improving well‐being outcomes, particularly for persons with problematic forms of smartphone use. This study assesses the effectiveness of an intervention app in enhancing well‐being outcomes and the moderating role of persons' perceptions about problematic smartphone use (PSU).

**Methods:**

In a randomized controlled trial, *N* = 70 participants, allocated to the intervention (*n* = 35) or control condition (*n* = 35), completed weekly online surveys at baseline, post‐intervention, and follow‐up. Participants from the intervention condition received personalized full‐screen nudges to reduce their social media app use. This secondary analysis focuses on the repeatedly assessed outcomes well‐being, positive affect, negative affect, and perceived stress. Linear mixed models were computed.

**Results:**

No significant time x group effects were found, but intervention condition participants reported higher well‐being and lower negative affect and stress levels at follow‐up. Only one significant moderation was found, indicating that participants reporting higher PSU levels benefited more from the intervention in reducing stress.

**Conclusions:**

The intervention was partly effective and particularly beneficial in reducing stress among smartphone users with higher PSU, highlighting the need to tailor interventions. Present findings need to be replicated by future research using a larger sample size.

## INTRODUCTION

With 4 billion active users worldwide and an average daily usage time of 2 hours and 24 minutes, social media use continues to rise (Statistical Research Department, 2024). Smartphones provide constant access to social media platforms, thereby facilitating various ways for individuals to communicate and interact (Brevers & Turel, 2019; O'Regan, 2016). Users create and share content, such as interests, images, or personal messages across their digital environment at any time and from any location (Kietzmann et al., 2011; Obar & Wildman, 2015). As social media becomes increasingly interwoven with users' daily lives, concerns about its potential negative effects on well‐being are rising, resulting in users actively seeking out strategies for digital disconnection (Jorge et al., 2022).

## REASONS TO DISCONNECT FROM SOCIAL MEDIA

A framework that aims to conceptualize the various ways users disconnect from digital technologies is the *process‐based framework of digital disconnection* (Vanden Abeele et al., 2024). The framework emphasizes the role of users making a conscious decision about their disconnection process and outlines several motivations for why users actively seek to digitally disconnect (Vanden Abeele et al., 2024).

One motivation for users to disconnect from social media is the feeling of wasting time online (Jorge et al., 2022). Social media features, such as an infinitely scrolling feed (Lyngs et al., 2022), can prompt compulsive usage patterns and challenge self‐control (i.e., the ability to override or change one's inner responses, as well as to interrupt undesired behavioral tendencies and refrain from acting on them; Tangney et al., 2004). By going down a rabbit hole, users are often unaware of the time passed while using social media (Horwood & Anglim, 2019; Schmuck, 2020). While high self‐control facilitates resistance to automatic and impulsive usage patterns (Brevers & Turel, 2019), low self‐control hinders disengagement, thereby fostering feelings of time displacement or wastefulness in online activities (Jorge et al., 2022). Consequently, social media use can displace rewarding activities, such as sleep or face‐to‐face interactions, which may serve as an essential source for well‐being (Kushlev et al., 2019; Reinecke et al., 2022).

Another motivation for users to digitally disconnect relates to the disruptive aspects of social media use in daily activities (Kushlev & Leitao, 2020). Short and frequent interruptions (i.e., direct messages or push notifications), can challenge users' attentional focus on primary tasks (e.g., work), leading to undesirable multitasking behavior and promoting feelings of overload (Aagaard, 2021; Elciyar, 2021). Moreover, users may not exclusively encounter entertaining social media content, such as funny cat videos; they might also come across harmful content that activates negative emotional arousal (Faelens et al., 2019). Particularly, social media platforms featuring high‐visual content, such as Instagram, can encourage social upward comparison (i.e., an idealized social media portrayal) and foster the need for validation, both of which negatively influence well‐being and drive users' need to disconnect (L. Faelens, Hoorelbeke, Cambier, et al., 2021; Meeus et al., 2019).

Digital disconnection is a motivated choice users pursue based on the perceived harms of usage and the potential benefits for well‐being (Vanden Abeele et al., 2024). Considering the dynamic nature of social media, where users select and are affected differently by social media use, seeking individualized strategies for digital (dis)connection becomes crucial (Vanden Abeele et al., 2024).

## DISCONNECTING FROM SOCIAL MEDIA

In recent years, a variety of intervention approaches have focused on digital disconnection as the main strategy to change usage behaviors (Nassen et al., 2023; Radtke et al., 2022). Some evidence suggests that social media disconnection has no effect on well‐being outcomes (e.g., Przybylski et al., 2021), whereas other findings indicate several benefits from social media disconnection, including an improvement in mental health and well‐being (e.g., Hou et al., 2019). Notably, interventions with a focus on changing usage behaviors through facilitating self‐control and reflection have shown promising effects for improving well‐being (Plackett et al., 2023).

While digital disconnection generally involves abstaining from or restricting the use of the digital device (Plackett et al., 2023; Radtke et al., 2022), technological intervention approaches have shifted their focus to supporting individuals in self‐regulating their social media use through so‐called *Digital Self‐Control‐Tools* (DSCTs) (Roffarello & Russis, 2023). DSCTs can be referred to as self‐binding applications with the focus on fostering more conscious and goal‐directed social media usage in alignment with users' personal goals and values (Monge Roffarello & De Russis, 2021; Roffarello & Russis, 2023). These tools mainly operate on the theoretical framework of nudging, which guides individuals' behavior towards a certain direction while retaining their autonomy of choosing freely (Thaler & Sunstein, 2021). DSCTs aim to provide supportive intervention content that is neither too restrictive nor easily ignored, including just‐in‐time approaches that intervene at the moment of overuse (Nahum‐Shani et al., 2018; Roffarello & Russis, 2023). In addition, some DSCTs include customizable features, based on self‐nudging to enable users to actively structure and design their intervention environment, for instance, by choosing their own time limit for specific apps (Reijula & Hertwig, 2022; Roffarello & De Russis, 2021). Research on the effects of interventions on social media use for well‐being outcomes shows inconsistent findings ‐ with full abstinence from social media even leading to negative consequences, such as cravings (Plackett et al., 2023; Radtke et al., 2022). Therefore, it might be essential to further explore tailored interventions that are not too restrictive and support users in self‐regulating their social media app use with the goal to enhance their well‐being.

## PROBLEMATIC SMARTPHONE USE

With the increasing use of social media, problematic smartphone use (PSU) has become a significant concern in today's digital age (Panova & Carbonell, 2018). PSU is characterized as a ‘person's inability to control one's smartphone use, resulting in impaired daily functioning’ (Elhai et al., 2017). The intensity of PSU can vary among individuals, with those experiencing high levels of PSU being particularly prone to excessive use of social media, often engaging in more prolonged and frequent usage sessions throughout the day compared to those who experience low levels of PSU (Pivetta et al., 2019; Świątek et al., 2023). In addition, high PSU is often linked to lower self‐control when it comes to social media usage, leading to behaviors such as compulsive checking or mindless scrolling, which are associated with adverse effects on well‐being (Augner et al., 2021; de Segovia Vicente et al., 2024). In the context of digital disconnection, users experiencing high PSU may benefit significantly from abstaining from social media. For instance, Turel et al. (2018) found that the interaction of social media disconnection and stress reduction was predicted by usage type, indicating that the intervention was more effective in reducing stress in heavy users compared to less heavy users. Similarly, Zhou et al. (2021) found that the effectiveness of their social media use intervention varied among users with different intensities of usage. While heavy users found it more difficult to abstain from social media, they also showed earlier and stronger improvements in life satisfaction and well‐being than normal users (Grüning et al., 2023; Keller et al., 2021).

Although research has continuously examined the link between PSU and well‐being outcomes (Elhai et al., 2017), little attention has been paid to the moderating role of PSU on the efficacy of interventions fostering well‐being (Plackett et al., 2023). Further research is needed to investigate whether an intervention aiming to self‐regulate social media use can enhance well‐being outcomes, particularly in users experiencing high PSU.

## THE PRESENT STUDY

This study investigates the secondary outcomes of a randomized controlled trial of a recently developed DSCT, the Wellspent app. The intervention app was developed to promote self‐regulated social media use on smartphones, which was based on a pilot study conducted between June and July 2022 (Keller et al., 2024). In the report on the primary outcome analysis of the present study (Mertens et al., in review), findings indicated significant improvements in the intervention (vs. control condition) in problematic smartphone use as well as significant reductions in time spent on participants' most problematic social media apps. However, no significant improvements in problematic social media app use and self‐efficacy were found (Mertens et al., in review). The intervention app is based on a self‐nudging and just‐in‐time approach (Nahum‐Shani et al., 2018; Reijula & Hertwig, 2022) and includes several behavior change techniques (Marques et al., 2024). The intervention app enables users to set personal time limits for self‐chosen problematic social media apps. The intervention app provides personalized, full‐screen nudges once a pre‐set usage limit is reached, encouraging users to end their current app session.

## AIMS AND HYPOTHESES

The present study aims to examine the effects of an intervention app (vs. control condition) targeting self‐regulated social media use for the secondary outcomes well‐being, positive affect, negative affect, and perceived stress. We expect that participants in the intervention condition (vs. control condition) will show an increase in well‐being (H = Hypothesis; H1a) and positive affect (H1b) as well as a reduction in negative affect (H1c) and perceived stress (H1d) over time. Based on previous findings of Turel et al. (2018) and Zhou et al. (2021) on the beneficial effects of digital disconnection for excessive social media and smartphone users, we expect stronger effects of the intervention on well‐being (H2a), positive affect (H2b), negative affect (H2c), and perceived stress (H2d) for individuals with higher PSU than for those with lower PSU.

## METHODS

### Sample and procedure

This study presents secondary analyses from a randomized controlled trial (RCT) testing an intervention app aiming to promote self‐regulated social media usage on smartphones in a convenience sample. The primary outcome analysis (Mertens et al., in review) has evaluated the effectiveness of the intervention app on problematic social media app use, PSU, and time spent on social media. The RCT was conducted between May and June 2023 and was approved by the Ethics Committee at Freie Universität Berlin. The RCT was preregistered at the German Clinical Trials Register (registration number: DRKS00031767).

Participants were recruited worldwide from the general population through social media platforms (Instagram, X, Facebook, LinkedIn) and university mailing lists. Eligibility criteria for participation included a minimum age of 18 years, proficient English language skills, regular usage of a smartphone with an iOS 16 operating system (because the intervention app was available for iOS systems only), and regular usage of at least one social media app on one's smartphone. The study participation was voluntary, with all participants signing an informed consent. The participation was also incentivized with a free premium one‐year subscription to the intervention app, which was offered by the collaborating partner Wellspent. In addition, psychology students at Freie Universität Berlin received course credit for full participation.

A total of *N* = 120 participants registered for the study between 4th May 2023 and 18th May 2023. Following registration, all participants were instructed to install the intervention app on their smartphones via a provided download link. Participants were then asked to set up the intervention app by selecting social media app(s) for which they intended to reduce their usage. In a baseline week, the app's intervention functions were deactivated for all participants, which included an empty home screen when opening the app. At the end of this week, participants responded to a baseline questionnaire (‘T' = Time; T1). Upon randomization after the baseline assessment, *n* = 50 participants had to be excluded as *n* = 33 did not download the intervention app and *n* = 17 did not complete the baseline assessment. Consequently, *n* = 70 participants (*n* = 47 female; 67%) with a mean age of 26.19 years (*SD* = 5.63; range 18–45) (see Table 1) were randomly assigned to an intervention (*n* = 35) and a control condition (*n* = 35). Randomization was facilitated using an online tool, similar to the procedure of ‘flipping a coin’ (Randomizer.org, n.d.).

**TABLE 1:** Demographic information about the total sample and per condition.

Variables	Total sample *N* = 70	Intervention condition *n* = 35	Control condition *n* = 35
Gender, *n* (%)			
Female	47 (67)	22 (63)	25 (71)
Male	23 (33)	13 (37)	10 (29)
Age, *M* (*SD*)	26.19 (5.63)	26.77 (6.56)	25.6 (4.53)
Nationality, *n* (%)			
German	56 (80)	28 (80)	28 (80)
American	3 (4)	2 (6)	1 (3)
Other nationalities	11 (16)	5 (14)	6 (17)
Education, *n* (%)			
Doctorate degree	3 (4)	1 (3)	2 (6)
Masters degree	13 (19)	8 (23)	5 (14)
Bachelor degree	23 (33)	10 (29)	13 (37)
Professional degree	4 (6)	2 (6)	2 (6)
Diploma	1 (1)	1 (3)	0 (0)
Trade	1 (1)	0 (0)	1 (3)
High school graduate	25 (36)	13 (37)	12 (34)
Employment, *n* (%)			
Student	39 (56)	21 (60)	18 (51)
Full‐time	20 (29)	10 (29)	10 (29)
Part‐time	8 (11)	2 (6)	6 (17)
Unemployed	1 (1)	1 (3)	0 (0)
Other	2 (3)	1 (3)	1 (3)

**FIGURE 1:**
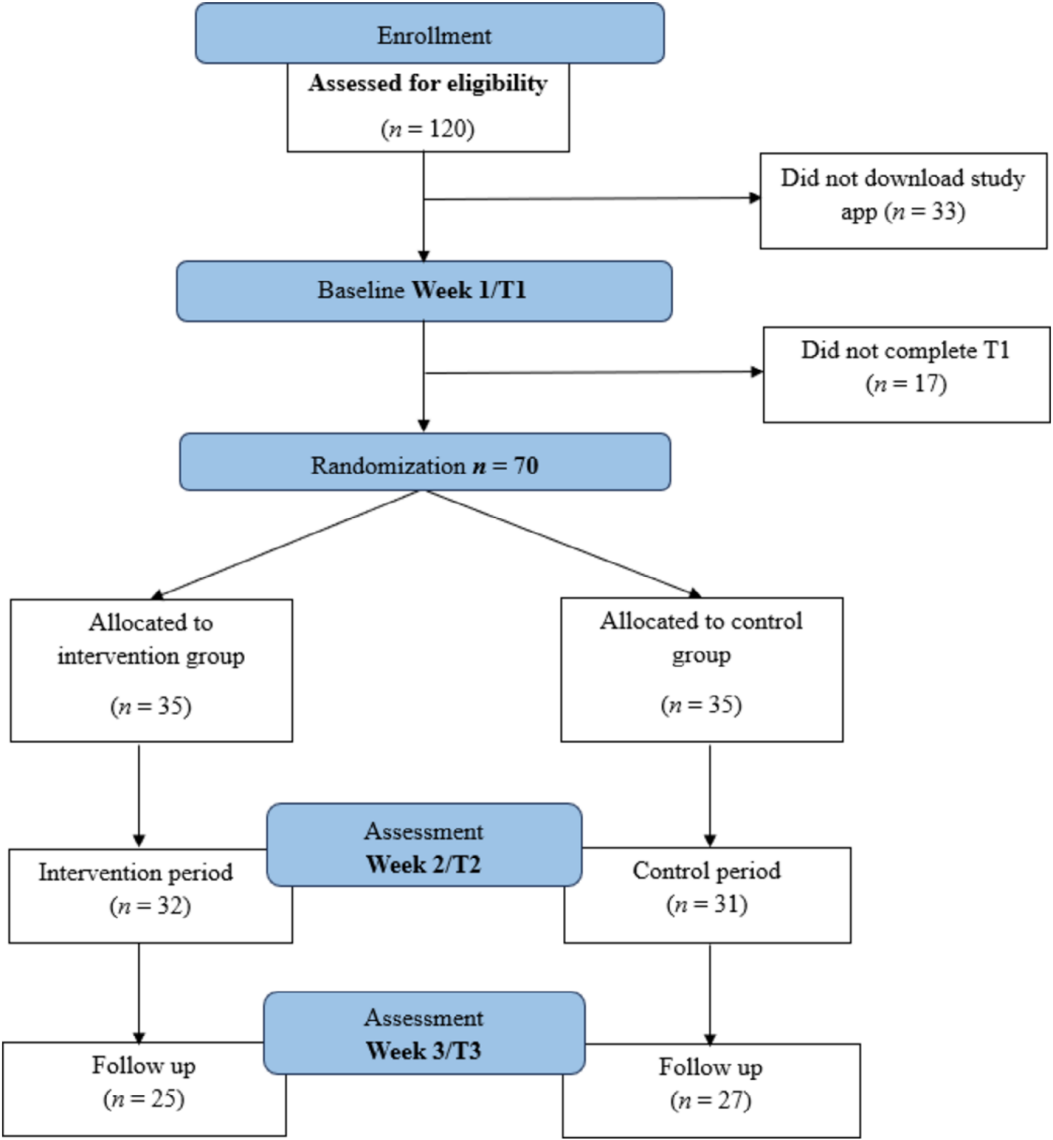
Flowchart showing condition allocation and participant dropout across the study. *Note*: ‘T' = time.

Following the baseline week, the intervention function of the app was activated for seven days for participants in the intervention condition only. The setup of the intervention app in the control condition remained as an empty home screen to the T3 assessment (i.e., the endpoint for secondary outcomes). At post‐intervention, participants in both conditions received a questionnaire at 7 days (T2) and 14 days (T3) following baseline (T1). Between T2 and T3, the usage of the app's intervention function was optional for participants in the intervention condition. Participants received reminders via email when responses were not initially provided to ensure continued participation. Participants in the control condition were given the opportunity to use the intervention app's function in Week 4. A further questionnaire at T4 (21 days following baseline) was provided. Present analyses focus on intervention effects between baseline and T3, which is the pre‐registered endpoint for secondary outcomes under study. A total of *n* = 52 participants from both conditions (out of 70: 74%) completed the follow‐up questionnaire at T3. In the intervention condition, *n* = 23 participants (out of 25 participants completing the T3 questionnaire: 92%) continued using the study app voluntarily during Week 3 (see Figure 1 for the CONSORT flowchart).

### Intervention

The intervention was implemented through the Wellspent app which includes a self‐nudging and just‐in‐time approach (Nahum‐Shani et al., 2018; Reijula & Hertwig, 2022) to promote self‐regulated social media use. When setting up the intervention app, participants were first asked to choose (a) social media app(s), for which they identified their usage as problematic (e.g., Instagram). Participants were then asked to set personal goals for the time spent on these apps using a customizable ‘budget' feature (e.g., 30 minutes per day) (BCIO 007004; Marques et al., 2024) and to specify the frequency of nudges they wish to receive (e.g., every 5 minutes). The app then offered participants to customize the intervention content to their individual app usage patterns. That is, participants identified their personal ‘danger zones’ (e.g., ‘I get lost on social media before bedtime’) and chose their preferred ‘tone of voice’ (e.g., humorous). They were also encouraged to define alternative activities they would prefer to engage in instead of spending time on social media (e.g., exercising).

When participants exceeded their self‐set nudge interval on their chosen social media app, the intervention app engaged with a full‐screen nudge. The full‐screen nudge provided feedback on the time the participant spent on the specific app (e.g., ‘You have spent 60 minutes on Instagram’; BCIO 007025; Marques et al., 2024) and suggested substitution of the social media behavior with the pre‐chosen alternative activity (e.g., ‘Let's go for a walk outside’; BCIO 007095; Marques et al., 2024). Further, the full‐screen nudge prompted participants to either quit or continue their current usage session. A detailed description of the nudges and personalized features is presented in Supporting Information S3.

### Measures

Self‐reported well‐being, positive and negative affect, and perceived stress were assessed at T1, T2, and T3 and referred to the past 7 days, respectively (see Table 2).

**TABLE 2:** Descriptive statistics of study variables for the intervention and control condition.

Study variables	Measurement occasion	Intervention condition		Control condition		
		*n*	*M (SD)*	*n*	*M (SD)*	
Well‐being [1–6]	T1	35	3.30 (0.93)	35	3.18 (1.04)	
	T2	31	3.55 (0.94)	30	3.21 (1.03)	
	T3	24	3.80 (0.78)	26	3.33 (0.97)	
Positive affect [1–5]	T1	35	2.23 (0.66)	35	2.39 (0.66)	
	T2	31	2.41 (0.68)	30	2.55 (0.64)	
	T3	24	2.48 (0.71)	26	2.72 (0.66)	
Negative affect [1–5]	T1	35	1.82 (0.61)	35	2.15 (0.82)	
	T2	31	1.83 (0.66)	30	2.34 (1.01)	
	T3	24	1.74 (0.71)	26	2.21 (0.91)	
Perceived stress [1–5]	T1	35	2.59 (0.80)	35	2.99 (0.96)	
	T2	31	2.44 (0.88)	30	2.94 (1.12)	
	T3	24	2.10 (0.74)	26	2.83 (0.94)	
Problematic smartphone use [1–5]	T1	35	3.00 (0.94)	35	3.03 (0.99)

*Notes*: T1: baseline. T2: intervention period. T3: follow‐up period. [1–6] and [1–5] refer to the response scale of the respective variable.

Abbreviations: *M*, mean; *SD*, standard deviation.

### Well‐being related to social media app use

Well‐being was assessed with an adapted version of the WHO‐5 Well‐Being Index (Brähler et al., 2007), contextualized to the usage of social media apps. Participants were instructed with ‘When thinking about your social media app use during the last 7 days, please indicate for each of the 5 statements how often you have been feeling that way’. Well‐being items (e.g., ‘In the past 7 days I felt cheerful and in good spirits’) were answered on a 6‐point scale ranging from (1 = *at no time*; 6 = *all the time*). Cronbach's alpha was α = .86 at T1, α = .90 at T2, and α = .83 at T3.

### Positive and negative affect related to social media app use

Self‐reported positive and negative affect were measured with an adapted version of the 10‐item *Positive and Negative Affect Schedule* (PANAS; Thompson, 2007). The items were contextualized to the context of social media usage. Participants responded to items such as ‘Please indicate the extent you have felt this way when thinking about your social media app use over the last 7 days’ on a 5‐point scale (1 = *very slightly* or *not at all*; 6 = *extremely*). Cronbach's alpha for positive affect was α = .69 at T1, α = .62 at T2, and α = .64 at T3. Cronbach's alpha for negative affect was α = .75 at T1, α = .84 at T2, and α = .84 at T3.

### Perceived stress

Perceived stress was assessed using an adapted version of the *Perceived Stress Scale* (PSS; Cohen et al., 1994; Murray et al., 2023) including 4 self‐reported items with responses ranging from ‘Never’ (1) to ‘Very often’ (5). A sample item read: ‘In the past 7 days, I felt that I was unable to control the important things in my life’. Cronbach's alpha was α = .83 at T1, α = .89 at T2 and α = .86 at T3.

### The moderator and covariates

The proposed moderator *problematic smartphone use* (PSU) was assessed at baseline (T1) using a one‐item self‐report. The item read ‘How often during the last 7 days have you found your smartphone use problematic?’ and was answered on a 5‐point scale (1 = *very rarely*, 5 = *very often*). In the study by Keller et al. (2021) the item correlated highly with the short version of the *Mobile Phone Problem Use Scale* (MPPUS‐10; Foerster et al., 2015). The covariates included sex (male = 0; female = 1), age, and education (0 = no university degree, 1 = university degree).

### Statistical analysis

The a‐priori power analysis was calculated using G*power v.3.1 (Faul et al., 2007) and included the following parameters for a repeated‐measures ANOVA: two groups, two measurement points (baseline and follow‐up), and a power of 1–*β* = .95. The analysis indicated that a longitudinal sample of 54 participants (27 participants per group) is needed to detect a medium‐sized time x group effect (*f* = .25) in the primary outcomes (Faul et al., 2007). It has to be noted that present analyses deviated from the ANOVA‐based power analysis because the use of multilevel modeling comes with the advantage of handling nested data structures, accounting for both within‐group and between‐group variability (Bolger & Laurenceau, 2013).

Secondary data analysis on intervention effects was based on the *intention‐to‐treat approach* (ITT). Rstudio with the lme4 package (Bates et al., 2015) was used to run multilevel models considering the nested data structure for repeated measurements T1, T2, and T3. We estimated four separate models for each outcome variable (well‐being, positive affect, negative affect, perceived stress), computing the interaction between experimental conditions (0 = control condition; 1 = intervention condition) and time (linear week trend; 0 = Baseline/Week 1, 1 = Week 2, 2 = Week 3). The linear mixed models were specified with a maximal random effect structure for all predictor variables (Barr et al., 2013), to account for both differences in respective outcomes (random intercept) and variability of changes over time (random slope of the time prediction) (Bolger & Laurenceau, 2013). Furthermore, we estimated four separate models for each outcome variable including PSU as the moderator, which was grand‐mean centered. The moderator's simple slopes were plotted at its mean and one standard deviation above and below its mean. To examine regions of significance, we conducted a simple slope analysis using the Johnson‐Neyman technique (Preacher et al., 2006). To attain missing data, a restricted estimated maximum likelihood (REML) method was applied (McNeish, 2017). Control variables age, positive affect, sex, and education were included as between‐person predictors. Age was grand‐mean centred while education and sex were dummy‐coded. All control variables were used for sensitivity analyses.

## RESULTS

### Randomization check and attrition analysis

We ran a randomization check for baseline variables using a binary outcome variable (0 = control condition, 1 = intervention condition). The randomization check revealed no between‐condition differences at baseline, indicating a successful randomization. In addition, an attrition analysis using chi‐square and t‐tests, followed by logistic regressions, was computed using a binary retainer variable (0 = non‐retainers; 1 = retainers). The attrition analysis showed that compared to participants retaining in the study (*n* = 52) participants who dropped out before T3 reported higher levels of positive affect (retainers: *M* = 2.20, *SD* = .67; non‐retainers: *M* = 2.59, *SD* = .53; *p* = .03).

### Changes in well‐being outcomes over time

In the first analysis, we aimed to test the intervention effect on well‐being (model 1), positive affect (model 2), negative affect (model 3), and perceived stress (model 4) up to a follow‐up at two weeks after baseline. The intraclass correlations (ICC) of weekly‐measured well‐being (ICC = .81), positive affect (ICC = .57), negative affect (ICC = .74), and perceived stress (ICC = .79) indicated that variance in outcome variables is mainly due to differences between participants (level 2).

Compared to time trends in the control condition of non‐significant changes for the outcomes in well‐being (*b* = .14, *SE* = .09, *p* = .13, *CI* 95% [−.04, .32]), negative affect (*b* = .11, *SE* = .09, *p* = .23, *CI* 95% [−.07, .29]), and perceived stress (*b* = −.11, *SE* = .09, *p* = .22, *CI* 95% [−.29, .07]), and slight increases in positive affect (*b* = .25, *SE* = .09, *p* = .01, *CI* 95% [.07, .43]), the following time x group interaction results were found. For the outcomes well‐being (*b* = .18, *SE* = .13, *p* = .20, *CI* 95% [−.09, .44]), positive affect (*b* = −.07, *SE* = .13, *p* = .60, *CI* 95% [−.33, .19]), negative affect (*b* = −.21, *SE* = .14, *p* = .13, *CI* 95% [−.47, .06]), and perceived stress (*b* = −.09, *SE* = .13, *p* = .51, *CI* 95% [−.36, .17]) non‐significant time × group interactions were observed (see Table 3).

**TABLE 3:** Estimates of two‐level model predictions of well‐being, positive affect, negative affect, and perceived stress, with covariates using the control condition as reference group.

Fixed effects	Model 1a: well‐being			Model 2a: positive affect			Model 3a: negative affect			Model 4a: perceived stress		
	Est (*SE*)	*p*	95% CI	Est (*SE*)	*p*	95% CI	Est (*SE*)	*p*	95% CI	Est (*SE*)	*p*	95% CI
Intercept at baseline	2.73 (0.44)	<.001	1.88, 3.56	2.25 (0.18)	<.001	2.15, 2.89	1.47 (0.35)	<.001	0.82, 2.13	2.44 (0.43)	<.001	1.62, 3.25
Time^a^	0.14 (0.09)	.13	−0.04, 0.32	**0.25 (0.09)**	**.01**	**0.07, 0.43**	0.11 (.09)	.23	−0.07, 0.29	−0.11 (0.09)	.22	−0.29, 0.07
Group^b^	**0.51 (0.23)**	**.03**	**0.08, 0.94**	−0.28 (0.18)	.14	−0.63, 0.08	**−0.57 (.22)**	**.01**	**−0.99, −0.14**	**−0.55 (0.25)**	**.03**	**−1.02, −0.07**
Time × group	0.18 (0.13)	.20	−0.09, 0.44	−0.07 (0.13)	.60	−0.33, 0.19	−0.21 (0.14)	.13	−0.47, 0.06	−0.09 (0.13)	.51	−0.36, 0.17
Age	0.02 (0.02)	.45	−0.03, 0.06	−0.01 (0.01)	.66	−0.04, 0.02	−0.05 (0.02)	.01	−0.08, −0.02	−0.02 (0.02)	.29	−0.07, 0.24
Sex^c^	−0.29 (0.23)	.21	−0.73, 0.15	0.33 (0.14)	.02	0.03, 0.60	0.44 (0.17)	.01	0.11, 0.77	0.62 (0.22)	.01	0.20, 1.03
Education	0.12 (0.25)	.63	−0.35, 0.59	0.09 (0.15)	.58	−0.21, 0.38	0.17 (0.18)	.36	−0.18, 0.52	−0.20 (0.23)	0.40	−0.65, 0.25
Positive affect	**0.34 (0.17)**	**.04**	**0.03, 0.65**				0.18 (0.12)	.15	−0.06, 0.41	0.03 (0.16)	0.86	−0.27, 0.32

Random effects (variances)	Model 1a: well‐being		Model 2a: positive affect		Model 3a: negative affect		Model 4a: perceived stress	
	Estimate	95% CI	Estimate	95% CI	Estimate	95% CI	Estimate	95% CI
Intercept	.89	0.68, 1.06	.51	0.30, 0.67	.72	0.49, 0.89	.84	0.58, 1.04
Time	.28	0.00, 0.45	.25	0.00, 0.42	.29	0.01, 0.45	.26	0.00, 0.45
Residual variance	.43	0.35, 0.53	.45	0.37, 0.54	.43	0.35, 0.52	.44	0.36, 0.53
ICC	.81		.57		.74		.79	
Pseudo‐*R* ^2^	.81		.58		.75		.81	

*Notes*: Models are based on data from *n* = 70 participants and *n* = 169 observations due to missing values. Coefficients smaller than .005 were rounded to .01.

Abbreviations: CI, confidence interval; Est, estimate; ICC, intraclass correlation.

^a^ Time coded −2 = T1, −1 = T2, 0 = T3.

^b^ Group is coded 0 = *control condition*, 1 = *intervention condition*.

^c^ Sex coded as 0 = *male*, 1 = *female*.

In terms of group differences at T3, significant between‐condition differences in well‐being (*b* = .51, *SE* = .23, *p* = .03, *CI* 95% [.08, .94]), negative affect (*b* = −.57, *SE* = .22, *p* = .01, *CI* 95% [−.99, −.14]), and perceived stress (*b* = −.55, *SE* = .25, *p* = .03, *CI* 95% [−1.02, −.07]), but no significant between‐condition differences for positive affect (*b* = −.28, *SE* = .18, *p* = .14, *CI* 95% [−.63, .08]) were found. The random effects of respective intercepts indicate significant between‐participant differences for the outcomes well‐being, positive affect, negative affect, and perceived stress. The random slope variances show that these outcomes change differently over time among participants (Bolger & Laurenceau, 2013).

In addition, we conducted a post‐hoc power analysis using RStudio and the simr‐package (Green & MacLeod, 2015). With the aim to find a time x group interaction of a small effect size (*f* = 0.19) using two‐level linear‐mixed models, considering the sample characteristics (2 groups, 3 measurement time points, *n* = 70), our present study lacks statistical power (i.e., .33).

### Moderation analysis

We also tested whether potential changes in the intervention condition (vs. control) for well‐being, positive affect, negative affect, and perceived stress were moderated by levels of problematic smartphone use at baseline. We found non‐significant moderation effects for well‐being (*b* = .06, *SE* = .15, *p* = .68, *CI* 95% [−.22, .35]) and positive affect (*b* = −.10, *SE* = .14, *p* = .47, *CI* 95% [−.38, .17]). A significant time x group x PSU interaction effect was found for the outcome negative affect (*b* = −.32, *SE* = .14, *p* = .03, *CI* 95% [−.60, −.06]), which was not confirmed in simple slope analyses (see Supporting Information S1).

**FIGURE 2:**
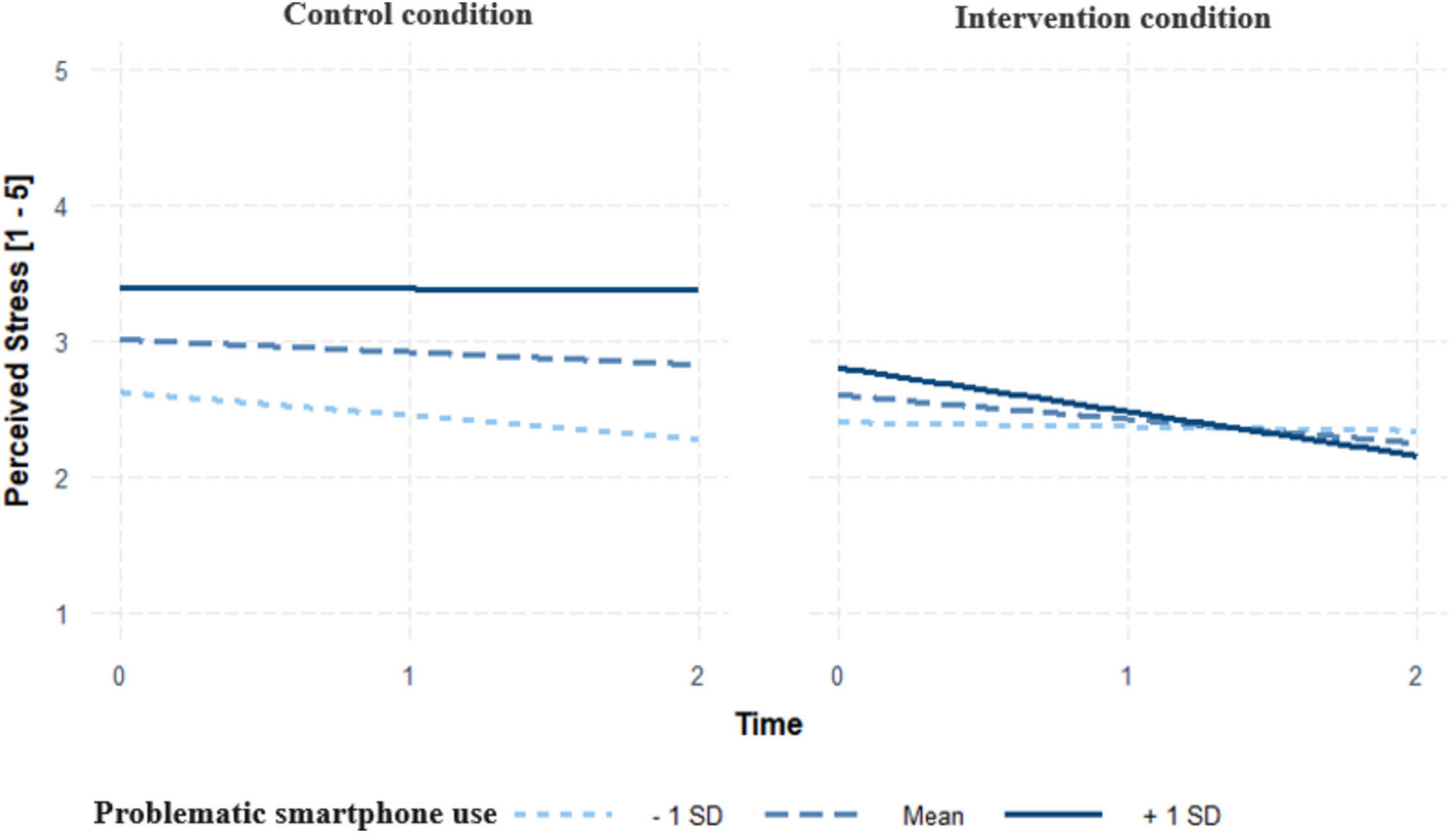
Relationship between the intervention (vs. control condition) and perceived stress for low (*M* – 1*SD*), average, and high (*M* + 1*SD*) levels of PSU. *Note*: Time coded as 0 = Baseline/Week1, 1 = Week 2, 2 = Week 3.

For perceived stress as the outcome, a relevant time x group x PSU interaction effect was observed (*b* = −.31, *SE* = .14, *p* = .03, *CI* 95% [−.58, −.04]). The simple slope analysis showed a decrease in perceived stress in intervention condition participants displaying higher‐than usual PSU (*b* = −.42, *SE* = .13, *p* < .001) and average PSU (*b* = −.23, *SE* = .09, *p* = .02). Changes over time in perceived stress were not statistically significant for participants from the intervention condition reporting lower‐than‐average PSU (*b* = −.04, *SE *= .14, *p *= .77). Post‐hoc analyses on regions of significance applying the Johnson‐Neyman technique illustrated that the intervention and perceived stress relationships were significant when between‐person centered PSU levels were above –0.18, which translates to PSU levels of 2.83 (see Figure 2).

.  

## DISCUSSION

As a secondary analysis of a randomized controlled trial, the present study examined the effectiveness of an intervention app (vs. control condition) targeting self‐regulated social media use for the outcomes well‐being (H1a), positive affect (H1b), negative affect (H1c), and perceived stress (H1d). Moreover, the moderation of intervention effects by different levels of PSU was investigated.

Not in line with our expectations, no intervention effect on time x group changes in well‐being (H1a) and positive affect (H1b) as well as negative affect (H1c) and perceived stress (H1d) were observed. However, we found significant group differences for the intervention (vs. control condition) with improved well‐being and reduced negative affect and perceived stress at T3. In addition, an increase in positive affect over time in both groups was observed. While these findings mirror the overall mixed results on the effects of digital disconnection interventions on improving well‐being outcomes (Plackett et al., 2023; Radtke et al., 2022), there are several possible explanations for the lack of intervention effects.

First, the relationship between social media disconnection and well‐being has been characterized as ambivalence (Ytre‐Arne et al., 2020). For example, Przybylski et al. (2020) found that the relationship between digital screen use and psychological functioning follows an inverted U‐shape, with excessive screen time leading to negative effects, but complete abstinence is also linked to negative consequences. In this regard, social media disconnection might have increased well‐being but simultaneously activated feelings of missing out, potentially attenuating the positive effects of disconnection (Hiniker et al., 2016). Further, inter‐individual variability in changes over time in well‐being outcomes emphasizes the importance of recognizing and considering differences between persons when evaluating interventions. Future studies could address this ambivalence by focusing on within‐person variations using a N‐of‐1 study design (Kwasnicka et al., 2019), investigating optimal levels of social media use and disconnection to enhance well‐being.

Secondly, our intervention app operates on self‐selected ‘time intervals’ (e.g., full‐screen nudge after ten minutes of TikTok usage), which might be potentially useful for investigating hypotheses linked to time‐related outcomes, such as procrastination or distraction (Aalbers et al., 2022; Siebers et al., 2022). Additionally, when focusing on well‐being outcomes, existing research suggests considering content predictors (Kross et al., 2021; Valkenburg, 2022). For example, studies have shown that certain social media content, such as pictures of thin bodies (Keipi et al., 2017), can be more harmful than others, such as pictures of puppies (Golbeck, 2019). It is possible that merely intervening on the duration of social media usage does not fully address all facets of the health‐compromising impact of social media use. A future study could explore adaptive content filtering that allows users to select and customize the type of content they wish to avoid, which can be adjusted dynamically based on user interactions and feedback (Burr et al., 2020).

Additionally, it is important to note that social media usage follows a fragmented nature, where multiple usage sessions are scattered throughout the day rather than occurring as a few prolonged usage periods (Deng et al., 2019; große Deters & Schoedel, 2024). Our study focused on weekly well‐being, affect, and perceived stress, while social media usage and thus the intervention might have occurred several times throughout the day. Similar to Stieger and Lewetz (2018), it might be crucial to account for the fragmented nature of usage sessions and implement a more detailed approach for evaluating the immediate effects of our intervention on well‐being outcomes through, for instance, situation‐specific assessments based on Ecological Momentary Assessment methods.

We found between‐group differences at T3 in favor of the intervention condition for the outcomes well‐being, negative affect, and stress. This finding points to partial effectiveness of the intervention app but is not in line with non‐significant time x group effects and should be interpreted with caution. Present analyses were powered to detect a medium‐sized time x group effect, thus, a small‐sized effect might not be detectable given the present sample size. Future studies should recruit and enroll a larger sample. Furthermore, we observed increases in positive affect in both study groups, which can be attributed to responding to the weekly questionnaires (MacNeill et al., 2016). Participants likely became more aware of their social media usage potentially leading to more positive or mindful interactions with social media.

## THE ROLE OF PROBLEMATIC SMARTPHONE USE

When examining the effects of social media use‐related interventions, the analysis of moderating factors is an important means to explore differences in effectiveness for sub‐groups (Vanden Abeele et al., 2024), such as users with higher or lower PSU. Users experiencing high PSU are assumed to be more negatively affected by social media (Elhai et al., 2017; Fabio et al., 2022), which is why we assumed that the intervention is more helpful and beneficial for individuals reporting higher PSU levels.

Not in line with our assumptions (H2a, H2b, H2c), the combination of the intervention app and high PSU levels did not result in increased well‐being and positive affect, nor in reduced negative affect. This is not in accordance with findings from Zhou et al. (2021) who reported that heavier social media users tended to show earlier and more pronounced improvements in well‐being when engaged with a similar intervention. However, the effectiveness of our intervention might be challenged by the enforcement level of the intervention app (i.e., the degree to which the app actively monitors, regulates, and restricts user behavior). It is possible that the present intervention app imposes a lower level of enforcement compared to interventions that implement restricted use or complete abstinence (Plackett et al., 2023). In addition, digital habits might be more ingrained in heavy users, taking a longer duration to achieve noticeable benefits (Hu et al., 2018). Addressing these challenges, future studies could extend the duration of the intervention period (i.e., one week) so that effects on well‐being outcomes can unfold over time. Consistent with our assumptions, the effectiveness of the intervention app in reducing stress was moderated by levels of PSU, with users experiencing average to high levels of PSU reporting a decrease in stress (supporting hypothesis H2d). Our findings align with existing research (Turel et al., 2018), suggesting that the more users perceive PSU and other compulsive usage patterns, the more they benefit from the intervention, whereas participants with low PSU report no reduced stress levels. By prompting users to set realistic usage goals and providing feedback on their social media behavior, the intervention app potentially helped in breaking cycles of compulsive behavior and reduced the overall stress associated with heavy social media use (Lyngs et al., 2019; Purohit & Holzer, 2021). In addition, by allowing users the autonomy of a free choice to either continue or quit their current social media session, our interventions app design likely facilitated an effective balance between connectivity and disconnection, a challenge often encountered in other digital disconnection studies, especially for users experiencing higher PSU (Hanley et al., 2019; Wilcockson et al., 2019). In this respect, our study expands on the process‐based framework of digital disconnection (Vanden Abeele et al., 2024) by offering the first evidence of moderating effects for the relationship between social media disconnection and well‐being, contributing to a deeper understanding of how DSCTs can effectively reduce stress in individuals reporting higher levels of problematic smartphone use. This highlights the importance of future studies to further investigate moderating and mediating variables (e.g., self‐regulation) when evaluating intervention effects of social media disconnection.

## STRENGTH, LIMITATIONS AND FUTURE DIRECTIONS

The present study has several strengths, including the study design as a randomized controlled trial, an innovative just‐in‐time approach, and a self‐nudging design, which leads to tailored and personalized intervention content, such as replacing social media time with reading or exercising, which differs from current screen time apps (e.g., iOS screen time).

However, some limitations need to be considered and adapted by future research. First, the sample size only provides sufficient statistical power to detect medium‐sized, but not low‐sized intervention effects, which may increase the likelihood of false negative results. Future studies should recruit a larger sample and include further strategies for reducing dropout (e.g., gamification elements). Second, our sample consisted exclusively of iPhone users and participants willing to change their social media use behavior. Notably, iPhone users are represented by individuals with a higher socio‐economic status compared to Android users (Rahmati et al., 2012). Future studies should enroll a representative sample, involving iPhone and Android users, to enable conclusions for the general population. Third, our study is based on retrospective self‐report data, which might be affected by recall biases (Tourangeau et al., 2000). While social media use follows a fragmented nature (Deng et al., 2019) and well‐being, affect, and stress might fluctuate throughout the day (H. Faelens, Hoorelbeke, Soenens, et al., 2021), it might be crucial to incorporate experience sampling methods, similar to Stieger and Lewetz (2018) or Cho et al. (2021). This would allow to examine the nuanced interplay between social media disconnection and well‐being outcomes. Fourth, our study did not use a context‐specific measure of perceived stress related to participants' social media app use, which may have underestimated present intervention effects on stress. Future research should consider additionally using context‐specific measures as it was recommended for various health contexts (Ambuehl & Inauen, 2022). Fifth, our intervention app intervened multiple times per day based on individuals' social media use, but it remained unclear whether participants performed another behavior instead of using their smartphone. To learn more about situational antecedents and behavioral changes, a future N‐of‐1 study could take a closer look and let people reflect on their social media use situations. Lastly, the three measurement points under investigation spanned three weeks, with the intervention app being active for one week. Although the study provides insights into the short‐time effectiveness, the brief duration may have limited the time needed for the intervention's effect to fully impact participants' well‐being in the medium‐ and long‐term, similar to findings in another app‐based intervention (Inauen et al., 2017). Future studies should also investigate the long‐term effectiveness of the intervention app for well‐being outcomes.

## CONCLUSION

Overall, our results mirror the mixed findings in the existing literature on digital disconnection interventions and their impact on well‐being outcomes (Plackett et al., 2023; Radtke et al., 2022). Additionally, our study underscores the potential of interventions to reduce perceived stress, particularly in users experiencing higher levels of PSU, which points to a promising direction for further research into personalized DSCTs. However, the mixed evidence on the effectiveness of the intervention app once more highlighted the balancing act between people's connectivity and disconnection. It also emphasizes that further research is needed to understand the complex interplay between content, duration, the context of usage, and a person's susceptibility regarding DSCTs or self‐nudging interventions (Vanden Abeele et al., 2024).

## CONFLICT OF INTEREST STATEMENT

LM was employed as a working student at the Wellspent GmbH during the time of the intervention period. CR is co‐founder of the Wellspent GmbH.

## ETHICS STATEMENT

The study was approved by the Ethics Committee of the Department of Education and Psychology at Freie Universität Berlin. All participants attended voluntarily and signed an informed consent.

## Supporting information


**Figure S1.** Relationship between the Intervention Condition (vs. Control Condition) and Negative Affect for very high (M + 2SD), high (M + 1SD), average, low (M – 1SD) and very low PSU (M ‐ 2SD).
**Figure S2.** Mean Levels over Time in the Control (blue) and Intervention (orange) Condition.
**Figure S3.** Intervention App Material, including Full‐Screen Nudges, Personal Budget, Tone of Voice, Goal Setting, and Habits.

## Data Availability

The research dataset is available from the corresponding author upon reasonable request.
