# Supplementary material for: Effects of an intervention targeting social media app use on well‐being outcomes: A randomized controlled trial
Source: Appl Psychol Health Well Being. 2025 Jan 24;17(1):e12646. doi: 10.1111/aphw.12646 (PMC11758673; doi:10.1111/aphw.12646)

**Supplementary Material**

**Effects of an Intervention Targeting Social Media App Use on Well-being Outcomes: A Randomized Controlled Trial**

**Content**

|  | **Content** | **Pages** |
| --- | --- | --- |
| S1 | *Relationship between the Intervention Condition (vs. Control Condition) and Negative Affect for very high (M + 2SD), high (M + 1SD), average, low (M – 1SD) and very low PSU (M - 2SD)* | 2 |
| S2 | *Mean Levels over Time in the Control (blue) and Intervention (orange) Condition* | 2 |
| S3 | *Intervention App Material, including Full-Screen Nudges, Personal Budget, Tone of Voice, Goal Setting and Habits* | 5 |

**Figure S1.**

*Relationship between the Intervention Condition (vs. Control Condition) and Negative Affect for very high (M + 2SD), high (M + 1SD), average, low (M – 1SD) and very low PSU (M - 2SD).*


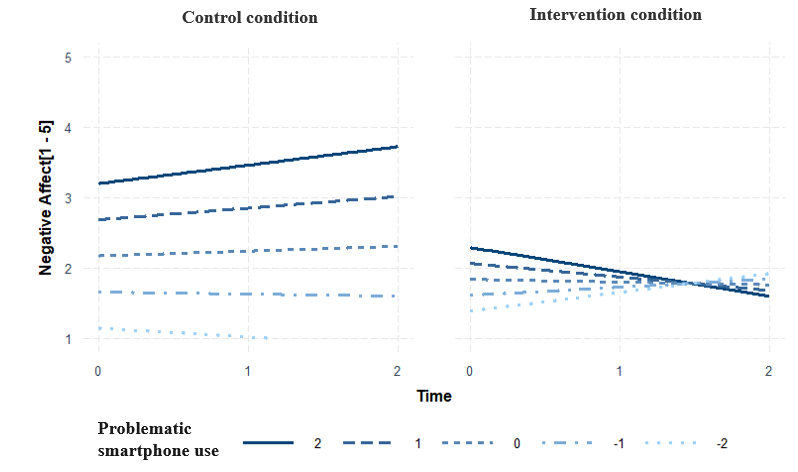


*Note.* The relationship between the intervention (vs. control condition) and negative affect for participants with very high PSU (*b* = -.45, *SE* = .23, *p* = .05), high (*b* = -.25, *SE* = .14, *p* = .08), average (*b* = -.05, *SE* = .09, *p* = .60) and low PSU (*b* = .15, *SE* = .15, *p* = .31). Post-hoc analyses on regions of significance using the Johnson-Neyman technique suggested that the relations of the intervention and reduced negative affect was marginal significant for PSU levels above – 0.45.

**Figure S2.**

*Mean Levels over Time in the Control (blue) and Intervention (orange) Condition.*

*Note.* Panels A, B, C, D only show sections of the response scale.

**Figure S3.**

*Intervention App Material, including Full-Screen Nudges, Personal Budget, Tone of Voice, Goal Setting and Habits*


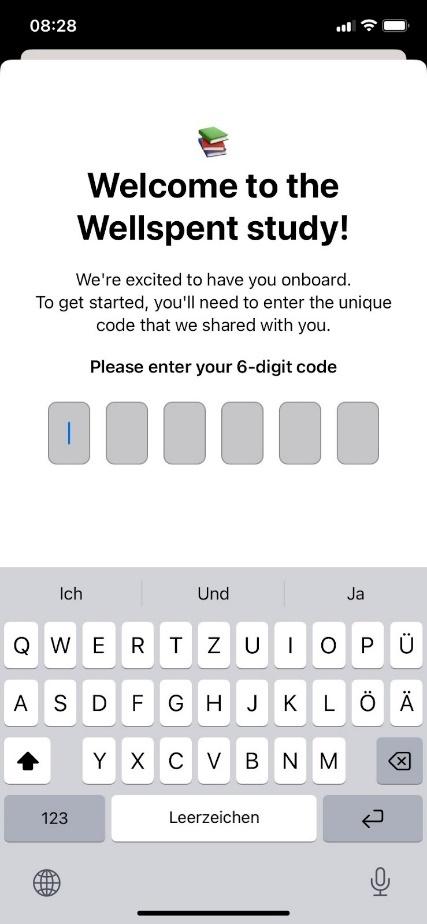

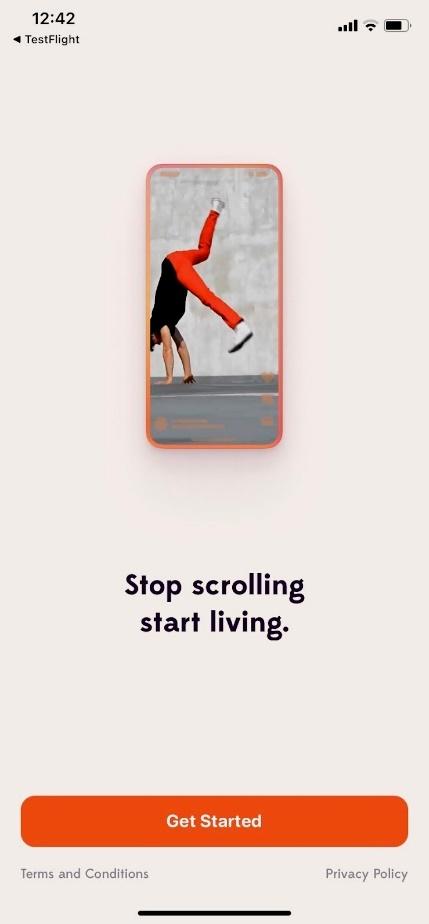

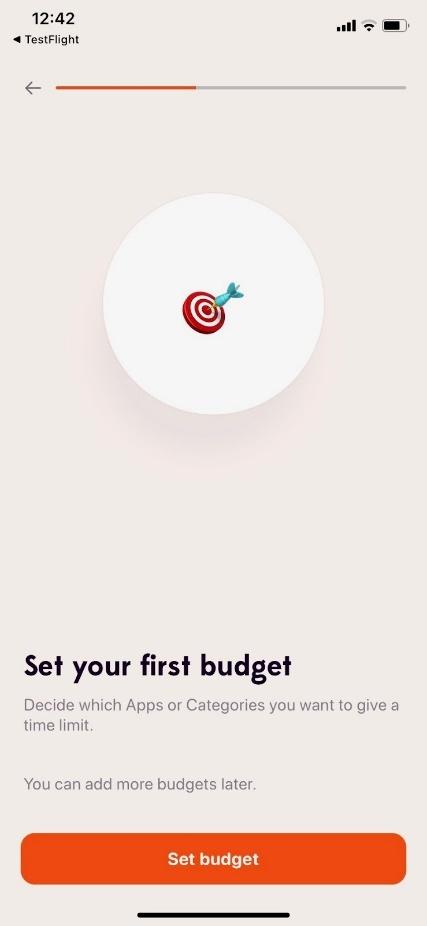

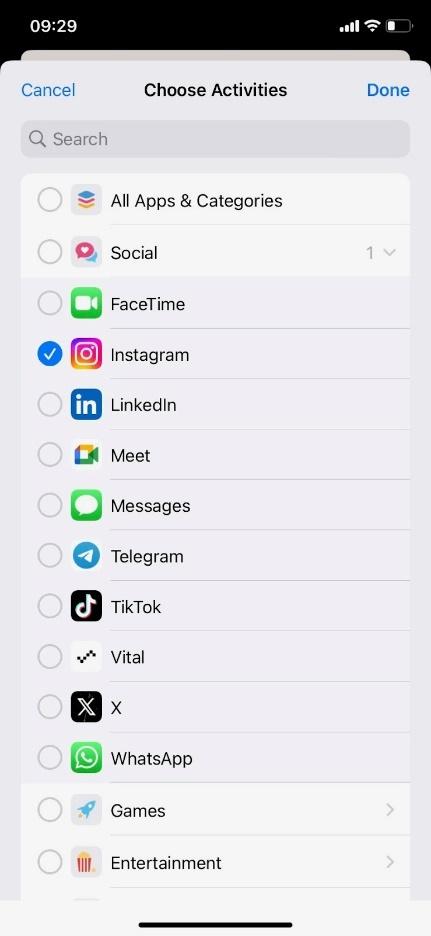

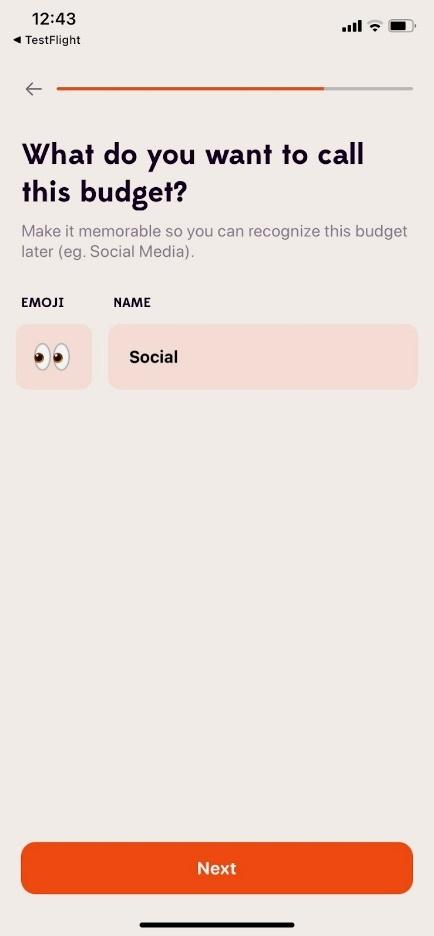

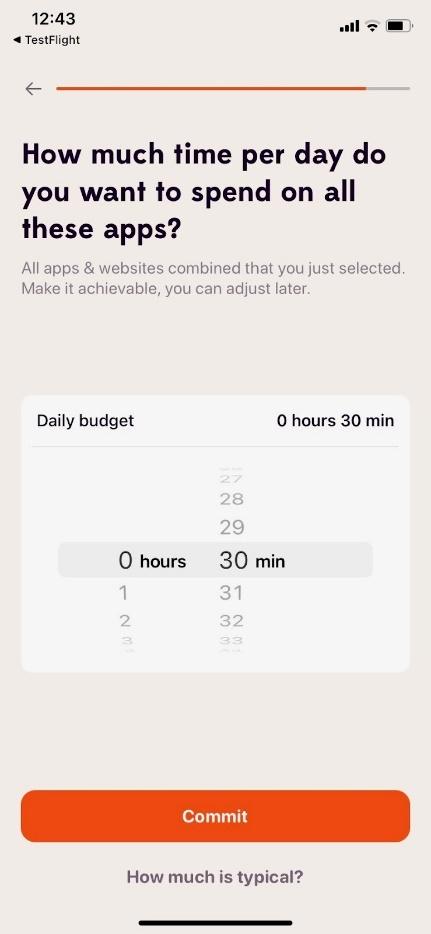


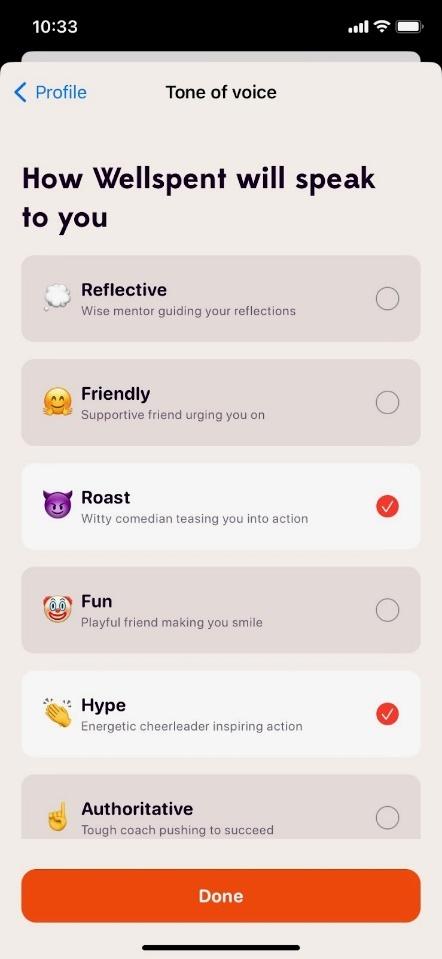

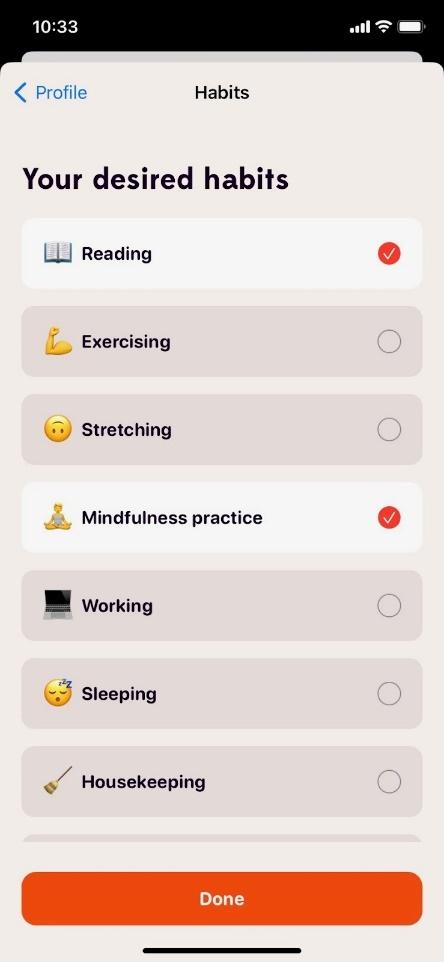

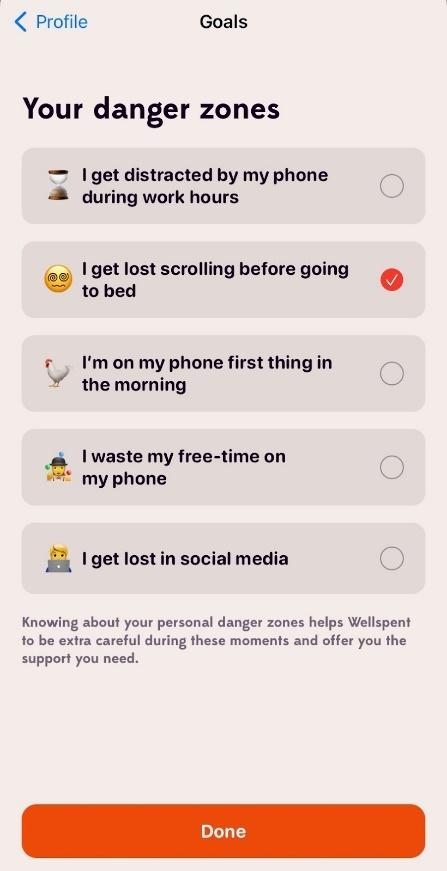

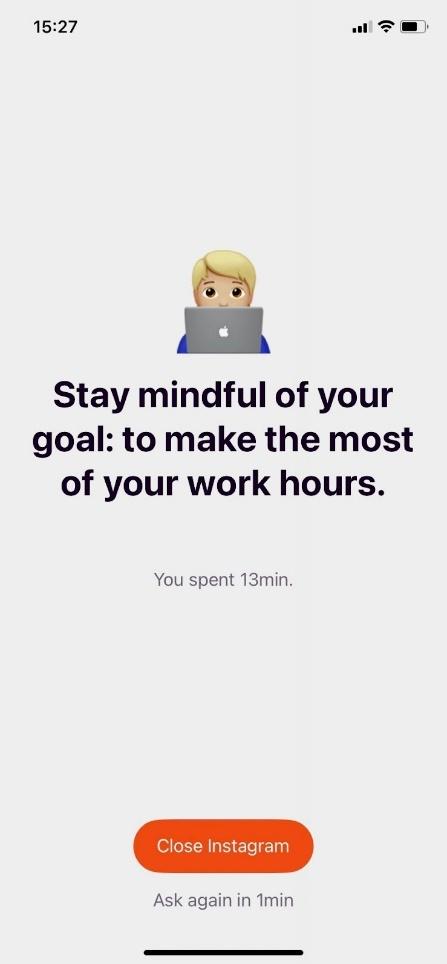

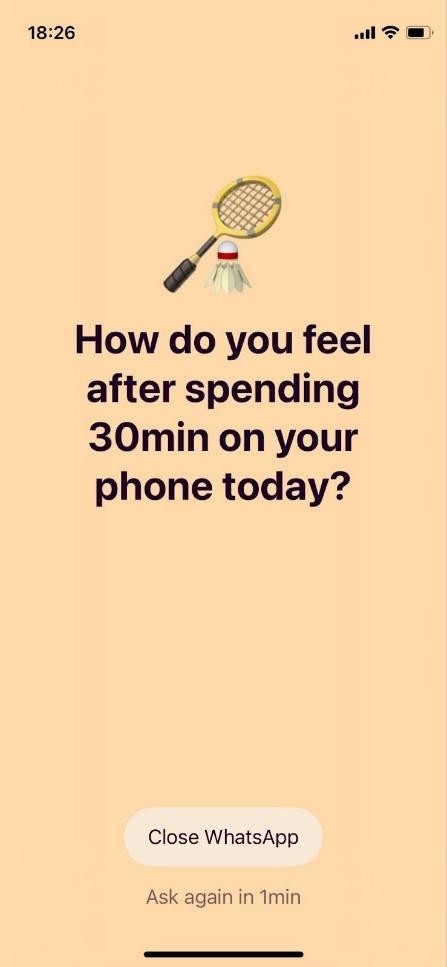

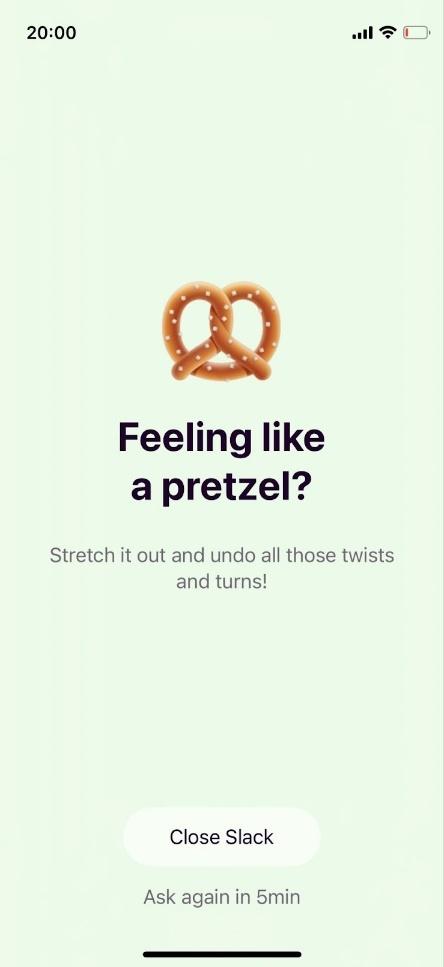


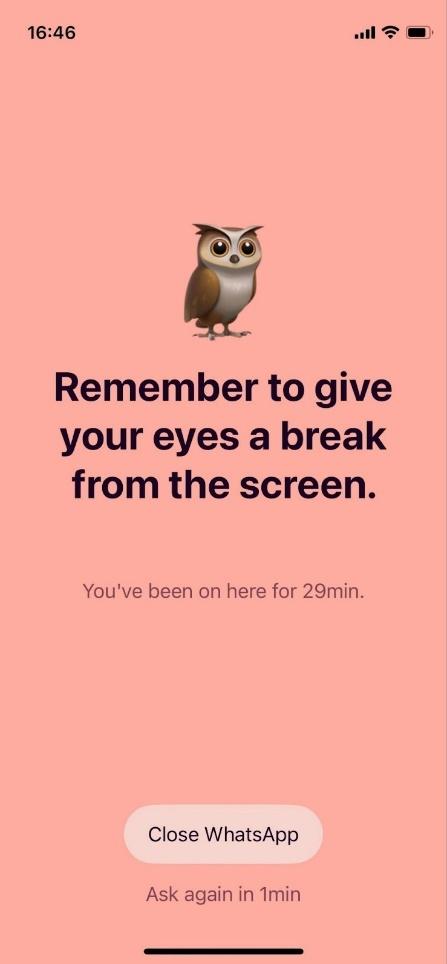

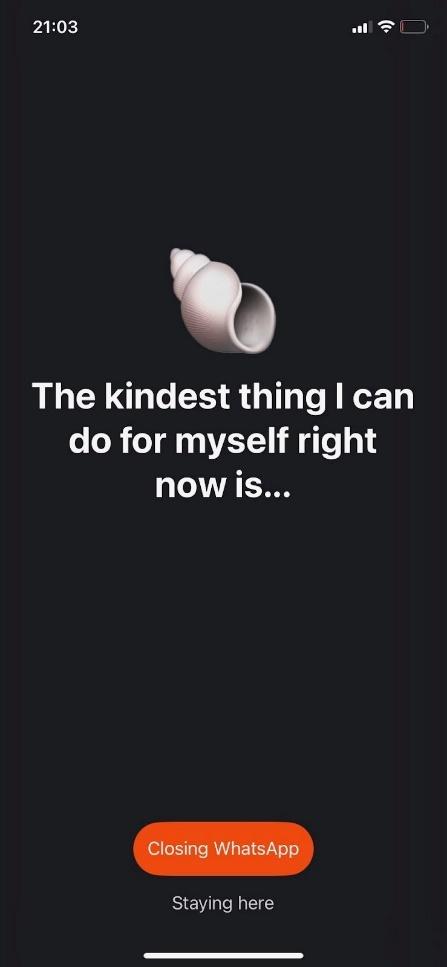

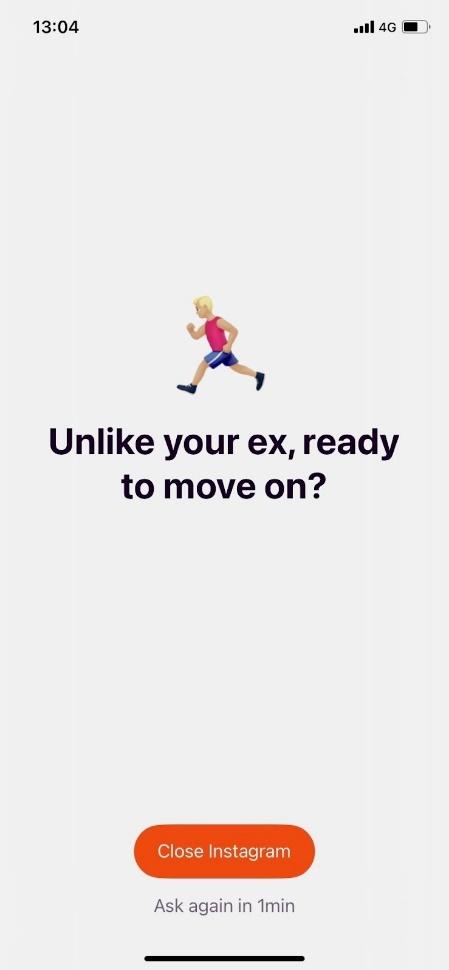

Supplement: Supplementary file 1 — Figure S1. Relationship between the Intervention Condition (vs. Control Condition) and Negative Affect for very high (M + 2SD), high (M + 1SD), average, low (M – 1SD) and very low PSU (M ‐ 2SD). Figure S2. Mean Levels over Time in the Control (blue) and Intervention (orange) Condition. Figure S3. Intervention App Material, including Full‐Screen Nudges, Personal Budget, Tone of Voice, Goal Setting, and Habits. [file APHW-17-0-s001.docx]
